# Scraps

**Published:** 1890-12

**Authors:** 


### SCRAPS

PICKED UP BY THE ASSISTANT-EDITOR.

**The Successful Treatment of Obesity.**—It is said that for this condition nothing is so efficacious as a few weeks' residence in a boarding-house where the person is "treated as one of the family."

**An Eye to Business.**—A certain doctor, who was noted for a keen eye to business, was driving along the street of a country town, when his horse took fright and ran away. He was thrown violently out of his trap and rendered senseless. Presently he recovered a little from his unconsciousness, and, noticing the crowd which had gathered about him, asked, "What's the matter, gentlemen? Anybody hurt? I am Dr. B. Can I be of any service?"

**Oriental English.**—The following testimonial was forwarded some time back to an English physician in the East:—"Dear Sir,—In July last my wife was down with fever and rheumatism of severest type, and I have to acknowledge with warmest and sincerest thanks the amiable and cordial assistance given by you in rescuing her at a time when all hopes of her existence were abandoned by one and all in the family, and even by professional men, who cried, '*Cui bono* are all our efforts?' You have attended on her without any torment, and without any exorbitant authoritative extortion, rather oppression, which some harpies doctors are apt to demand at such a critical juncture, and treated her gratuitously, harmonised with your native English, and unanimodes, and with such patience and diligence as the occasion demanded. Before concluding, I beg to state I am bidding my beads, and pattering my fervent prayers to the Almighty God to vouchsafe you with longevity, health, wealth, and prosperity."

**Medical Libraries.**—In view of the library which the Bristol Medico-Chirurgical Society has decided to establish for the use of its members, the following extracts from speeches made at the opening of the new building, which is to form the home of the New York Academy of Medicine, will be of interest The building in question is to furnish accommodation for a large library, with reading-rooms and commodious meeting-rooms for medical societies. Dr. Alfred L. Loomis, in the course of the president's address, said that, with a library capacity for 200,000 volumes and a well-selected library of 50,000 volumes, supplemented by the largest and best collection of journals to be found in that country, they might rightfully maintain that they were in some degree meeting the highest requirements of scientific medicine. The influence which such a library would have, not only on the intellectual status and culture of the profession, but upon its moral tone, could not be estimated: for there is an atmosphere about a large and well-selected library which does not favour the growth of a mean, money-calculating spirit; it conduces to broadness, tolerance, and a love of the higher and nobler attributes of man. Dr. Edward L. Keyes, in the Anniversary Oration, said that the library was founded by donations in 1877, and never bought a book till 1879, eleven years ago; yet now, safely housed in a fire-proof house, they were proud in possessing the third place numerically among the medical libraries of America, and the fourth place, as far as he could learn, among the purely medical libraries of the world. Dr. A. Jacobi said that thirty-three years ago there was no library at all, not even a medical reading-room in the city. It took many years before the Journal Association was organised, which furnished in a room fitted up for the purpose at No. 64 Madison Avenue, the current medical journals. Occasionally a sum was raised by voluntary contributions, for the purpose of buying the collection of a deceased member, certain publishers would present us with their publications, authors donate copies of their writings, fellows and others give old and new books, and men interested in special branches of literature furnish a shelf-ful of special works. That was their library. Thus it grew, slowly but steadily. The library, the representative and exponent of all medicine, is no longer a mere collection, but a vitalised organism. That is why there is an atmosphere of solemnity in a large library; for we are standing in the presence of the spirit and soul of all previous ages, each evolving from and connected with its neighbour. That is why a library is to the scientist what the church is to the pious. No consideration of lucre invites us there. While nourishing our minds, we disconnect ourselves from the embarrassments of trivial employment and deliver ourselves from the merely terrestrial. In that way the idealism is nurtured that no feeling and thinking man is to be without; idealism, without which no nation can expect to live. When she lost it, even Hellas perished, though she had given birth to Solon, Pericles, Aristides, and Sophocles. Dr. John S. Billings said that in the library we may at all times counsel with the wise and illustrious of our profession of all times and countries, living and dead, upon whatever subject most interests us. We can always find good company there; they are never in a hurry, never obtrusive, and while they cannot always tell us what we want to know, they can usually tell us something new that we ought to know, provided we question them aright. There should be a large expenditure upon periodical literature; for it is the papers and figures in these which the physician most frequently wishes to use to enable him to solve his own problems or to instruct others. Every physician should take five journals. Every medical teacher and every specialist should take a dozen. Every Medical Journal Club and small library should take from 25 to 50; every large medical library should take from 150 to 300; and the national collection in each country had better take them all, say 1000 or more. For journals, transactions, and reports, the physician must largely rely upon some general library. (For full reports of the opening reception see *New York Medical Journal*, November 22nd and 29th, 1890.)

Although this new departure of the New York Academy has been possible with a membership of less than 700, not even the most sanguine can hope that the Bristol Society can do anything approaching it. But, with co-operaticn and public spirit, the recent resolve to have a local medical library might result in incalculable good to the whole profession of the neighbourhood. The example of others who felt, as we feel, the want of a representative collection of medical works, and who, from a beginning certainly not larger than ours, have done so well, should stimulate us to make a strong and united effort to obtain that which will go a considerable way to meet some of our needs. This library of the Bristol Medico-Chirurgical Society deserves the liberal support of the local profession; for if aided, as it might be, by amalgamation with the Medical School, which now seems within measurable distance of getting its much-needed new buildings, it might lead to our possessing at no distant date a home which shall be the centre of medical life for this neighbourhood.

